# The effects of textured insoles on quiet standing balance in four stance types with and without vision

**DOI:** 10.1186/s13102-019-0117-9

**Published:** 2019-04-04

**Authors:** Ryan P. W. Kenny, Daniel L. Eaves, Denis Martin, Anna L. Hatton, John Dixon

**Affiliations:** 1 School of Health and Social Care, Teesside University, Middlesbrough, TS1 3BX UK; 2 School of Health and Rehabilitation Sciences, The University of Queensland, Brisbane, AU Australia

**Keywords:** Static balance, Footwear intervention, Sensory reweighting, Somatosensory input, Mechanoreceptors

## Abstract

**Background:**

Wearing a textured shoe insole can decrease postural sway during static balance. Previous studies assessed bipedal and/or unipedal standing. In contrast, we aimed to investigate if textured insoles modulated postural sway during four stance types (bipedal, standard Romberg, tandem Romberg, and unipedal), with and without vision.

**Methods:**

The repeated measures design involved 28 healthy young adults (13 females; mean age = 26.86 ± 6.6 yrs) performing quiet standing in the four stance types on a force platform, under two different insole conditions (textured insole; TI vs. smooth insole; SI), with eyes open and eyes closed. Postural sway was assessed via the range and standard deviation of the COP excursions in the anterior-posterior and medial-lateral sway, and overall mean velocity.

**Results:**

The main effect of insole type was statistically significant at the alpha *p* = 0.05 level (*p* = 0.045). Compared to smooth insoles, textured insoles reduced the standard deviation of anterior-posterior excursions (APSD). While simple main effect analyses revealed this was most pronounced during eyes closed bipedal standing, insole type did not provide a statistically significant interaction with either stance or vision in this measure, or any other. Postural sway showed statistically significant increases across both stance type (bipedal < standard Romberg < tandem Romberg < unipedal), and vision (eyes closed < eyes open), in almost all measures. Stance and vision did have a statistically significant interaction in each measure, reflecting greater postural disturbances with eyes closed when stance stability decreased.

**Conclusions:**

Overall, these results support textured insole use in healthy young adults to reduce postural sway measures. This is because APSD is an index of spatial variability, where a decrease is associated with improved balance and possibly translates to reduced falls risk. Placing a novel texture in the shoe presumably modulated somatosensory inputs. It is important to understand the underlying mechanisms by which textured insoles influence postural sway. As such, utilising a healthy adult group allows us to investigate possible mechanisms of textured insoles. Future research could investigate the potential underlying mechanisms of textured insole effects at a neuromuscular and cortical level, in healthy young adults.

## Background

Wearing a textured insole (TI) in the shoe can decrease postural sway during static balance tasks [1, 2] in a range of populations, including: healthy older people [3, 4], people with Parkinson’s disease [5], multiple sclerosis [6], and even healthy young adults [4, 7]. One proposal is that TIs improve balance by altering sensorimotor inputs via mechanoreceptors on the plantar surface of the feet [8]. Other studies show, however, that TIs do not always improve balance [9–13]. This discrepancy could be due to multiple factors, such as insole characteristics (including geometric patterns and material properties) [8], and whether different textures interact with stance type (which modulates balance difficulty), vision, and the sensorimotor pathologies of different populations. Next, we briefly explain our selection of a pyramidal shaped insole texture, before reviewing literature justifying our experimental manipulations.

A range of insole textures have previously been studied. Improvements in postural control during quiet standing balance have been obtained using a convex texture in people with Parkinson’s disease [5], and a spiked texture in healthy young and older participants [14, 15]. In comparison, no beneficial effects were observed when wearing a rounded nodule pattern in healthy young adults [9], and a spiked pattern in those with chronic ankle instability [11]. The more commonly used pyramidal patterning has also produced no improvements for static balance in middle-aged women [10], older adults with a history of falls [8], and in people with multiple sclerosis [12]. Conversely other studies have shown pyramidal patterning has generated positive effects in healthy young adults [7], healthy older people [4], and people with multiple sclerosis [6]. Hatton and colleagues [4, 7] also found advantages for a pyramidal compared to a concave textured floor pattern in young healthy adults and older people. Given these predominantly positive results, particularly in healthy young adults, we compared the pyramidal to a smooth insole (control) in the current study.

Previous static balance research has typically evaluated TI effects only in bipedal and/or unipedal quiet standing [7, 11, 12]. While earlier studies have manipulated balance difficulty, for example using foam vs. firm surfaces [4, 5], no previous studies have explored TI effects across a range of stance types that pose increasing challenges to postural stability. This approach could establish a profile for TI effects across multiple stance types and may thus serve to better inform subsequent interventions. We therefore manipulated the base of support to assess TI effects throughout a linear increase in task difficultly, from bipedal (feet apart) to standard Romberg (feet together), to tandem Romberg (feet heel-to-toe), to unipedal standing.

Since vision is predominantly involved in balance control [4], it is useful to isolate TI effects from the contribution of visual perception during quiet standing. TI studies have previously manipulated eyes open (EO) vs. eyes closed (EC), with some only observing a significant TI effect during EC [3, 4, 6], and others no effect during EO [7, 9]. A greater reliance on somatosensory information is likely required during EC, causing sensory reweighting: a phenomenon by which the relative contribution of each sensory system changes depending on environmental constraints [16]. In the absence of vision, we therefore predicted an increase in the magnitude of the TI effect due to sensory reweighting that emphasises proprioceptive sources. The present study is therefore the first to assess TI effects across four stance types, with and without vision.

The aim of the present study was to investigate the effect of TIs in multiple stance types, with and without vision. Overall, we hypothesised postural sway would be altered when wearing TIs compared to the smooth insole in each stance type. In addition, it was expected that stance and vision would modulate postural sway measures.

## Methods

### Participant information

A convenience sample of healthy young adults (*n* = 28; 13 females; mean age = 26.86 ± 6.6 yrs.; height = 171.82 ± 9.46 cm; weight = 73.22 ± 17.02 kg) volunteered, who were aged 18-51 years [1], right-footed, injury-free and with an adult shoe size (3–12, UK). Participants were excluded if they had neurological or musculoskeletal disorders, needed mobility aids, were injured or pregnant, had communicable foot diseases, or were unable to provide informed consent. Ethical approval was granted by the School of Health and Social Care Research Governance and Ethics Committee at Teesside University. Informed consent was obtained from all participants prior to testing.

### Design

The study involved a three-factorial repeated measures design comprising of insole type (textured vs. smooth), stance type (bipedal vs. standard Romberg vs. tandem Romberg vs. unipedal), and vision type (EO vs. EC), administered in a fully-randomised order. The dependent variable was movement of the centre of pressure (COP) during 30s of quiet standing, comprising five parameters: mean sway range (mm) and standard deviation (SD) in both anterior-posterior (AP range, APSD), and medial-lateral (ML range, MLSD) directions, and overall mean sway velocity (mm/s) of the AP and ML data.

### Materials

Two types of prefabricated insole were used, which have been investigated in previous studies [7, 12]. The upper surface of the TIs comprised small pyramidal peaks with centre-to-centre distances of approximately 2.5 mm (Evalite Pyramid EVA, 3 mm thickness, shore value A50; Algeos UK ltd.), cut to a range of men’s and women’s UK shoe sizes. The smooth insole (SI) acted as the control, having a completely flat upper surface (medium density EVA, 3-mm thickness, shore value A50; Algeos UK Ltd., Liverpool, UK). During the testing procedures the insoles were worn within a standardised shoe (rubber sole with upper canvas). Participants wore thin standardised socks for hygiene purposes.

Quiet standing balance was assessed using two Kistler force plates (Model: 9286AA, Kistler Instruments Ltd., Hampshire, UK), placed side-by-side and combined to make one plate (approximate 5 mm space between plates) via an integrated charge amplifier (Model: DAQ 5691, 16ch, Kistler Instruments Ltd.), sampling at 50 Hz. Two force plates were needed due to the tandem stance length (heel-to-toe) [17].

### Procedure

Leg dominance was assessed by watching participants kick a football [18]. Each condition (*n* = 16) consisted 5 × 30s balance trials, producing 80 trials per participant. Stance position was standardised on the force plates for each participant. During bipedal standing, participants were instructed to “stand in a position comfortable for you”. In standard Romberg participants stood with the medial arches of the feet together. The tandem Romberg stance was completed heel-to-toe, right foot forward [17]. Unipedal standing was performed on the dominant leg, since no differences exist between legs in this population [19]. During all stance types participants were instructed to stand as still as possible. During EO, participants looked at a target on a wall 3 m ahead of the force plates [7]. Trials commenced once participants adopted the standardised position (trunk erect, arms by sides, lower limbs extended). For EC trials participants closed their eyes beforehand. There was a 2 min seated break between stance conditions. All data was collected within a single testing session to assess acute effects of insole usage. Acute effects are important and commonly used because they provide the opportunity to assess the body’s initial response to changes in sensory input during balance (c.f., [3–5, 7, 9]). They also afford the groundwork for future studies to assess longitudinal effects. They also afford the groundwork for future studies to assess longitudinal effects. In addition, safety of participants must be considered. By assessing acute effects in a laboratory setting we can monitor participants responses to an unfamiliar device, prior to exploring any longitudinal effects, whereby participants are not within the constraints of a supervised laboratory setting.

### Data extraction

All COP variables were extracted from the force platform using Bioware software (Bioware V5.3, Kistler). All data processing was performed off-line using a commercial software package (MATLAB 2016a, The MathWorks Inc., Natick, MA). Since literature suggests 20–30s of data collection is sufficient to assess quiet standing [20], the minimum trial duration included for analysis was 20s. In bipedal and standard Romberg all participants successfully completed 30s quiet standing balance. In tandem Romberg and unipedal stances all participants completed 20–30s, with the majority of trials lasting 30s (*n* = 96.1 and 71.4%, respectively). Removed trials involved one foot stepping out of the required stance.

### Statistical analysis

A three-factorial repeated measures ANOVA was used to investigate the factors of insole type (smooth vs. textured), stance type (bipedal vs. standard Romberg vs. tandem Romberg vs. unipedal) and vision type (eyes open vs. eyes closed) in each of the five COP variables. Analyses were conducted using SPSS Statistics 23 (IBM, Chicago, IL, USA). We adjusted for any violation of the homogeneity of variance assumption using the Greenhouse–Geisser correction. Alpha levels were set to 0.05, and effect sizes were calculated as partial eta squared values (${\eta}_p^2$). Simple main effect analyses with Fishers LSD were used as post-hoc tests.

## Results

### Insole type

The main effect of insole was significant in the APSD variable, *F*(1, 27) = 4.38, *p* = 0.045, ${\eta}_p^2$ = 0.14. Post-hoc tests revealed APSD was significantly reduced in the textured, compared to the SI condition (6.92 ± 2.16 mm vs. 7.14 ± 2.51 mm, respectively; mean difference of 0.22 mm, 95% CI: 0.004 to 0.44). Simple main effect analyses investigating each of the four stance types in the APSD data showed postural sway was only significantly reduced in the textured compared to the smooth condition in the bipedal stance, *F*(1, 27) = 5.84, *p* = 0.02, ${\eta}_p^2$ = 0.18 (4.29 ± 1.32 mm vs. 4.8 ± 2.29 mm; mean difference of 0.51 mm, 95% CI: 0.076 to 0.935). While this pattern was replicated in both the standard and tandem Romberg stances, the associated alpha levels were not significant, see Table 1. More focused simple main effect analyses on the bipedal data revealed postural sway was significantly less for the textured compared to smooth insole in the EC condition, *F*(1, 27) = 4.68, *p* = 0.04, ${\eta}_p^2$ = 0.15 (4.88 ± 6.62 mm vs. 5.46 ± 6.6 mm; mean difference of 0.58 mm, 95% CI: 0.03 to 1.14), and was close to significant in the EO condition, *F*(1, 27) = 3.41, *p* = .076, ${\eta}_p^2$ = 0.11 (3.71 ± 4.12 mm vs. 4.14 ± 1.79 mm; mean difference of 0.43 mm, 95% CI: -0.047 to 0.901). The main effect of insole type was not significant in the other four COP measures: AP range, *F*(1, 27) = 0.78, *p* = 0.39, ${\eta}_p^2$ = 0.03, MLSD, *F*(1, 27) = 0.03, *p* = 0.87, ${\eta}_p^2$ < 0.01, ML range, *F*(1, 27) = 0.80, *p* = 0.38, ${\eta}_p^2$ = 0.03, and overall mean velocity, *F*(1, 27) = 1.71, *p* = 0.20, ${\eta}_p^2$ = 0.06.

**Table 1:** Mean (SD) APSD (mm), mean difference (95% CI), and alpha levels for stance and insole type

Stance	Mean SI (SD)	Mean TI (SD)	Mean dif. (95% CI)	*P*-Value
Bipedal	4.8 (2.29)	4.29 (1.32)	- 0.51 (−0.94 to − 0.08)	0.02
Standard Romberg	5.43 (1.84)	5.2 (1.63)	- 0.23 (−0.61 to 0.15)	0.23
Tandem Romberg	8.56 (4.83)	8.28 (4.32)	- 0.28 (−0.9 to 0.34)	0.36
Unipedal	9.76 (2.02)	9.88 (0.48)	0.12 (−0.29 to 0.54)	0.54

*SI*. smooth insole, *TI* textured insole, *SD* standard deviation, *dif.* difference, *CI* confidence interval

### Stance type

**Fig. 1:**
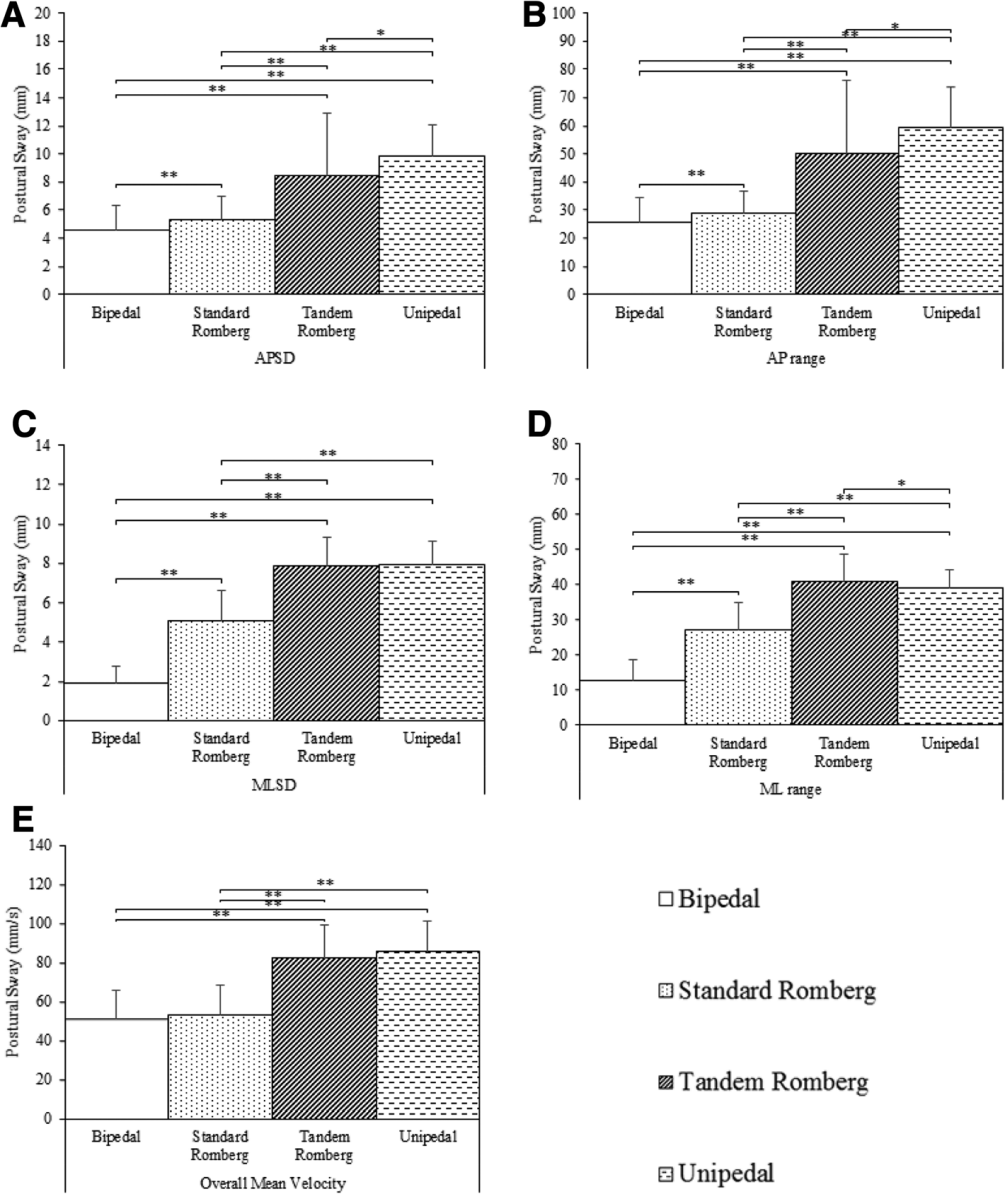
Mean postural sway in the five COP measures for the factor of stance. **a.** Mean postural sway for anterior-posterior standard deviation (APSD) (**b).** Mean postural sway for anterior-posterior range (AP range) (**c).** Mean postural sway for medial-lateral standard deviation (MLSD) data. **d.** Mean postural sway for medial-lateral range (ML range) data. **e.** Mean postural sway for the mean velocity data. ** denotes a significant difference of *p* < .001. * denotes a significant difference of *p* < .05. Error bars show standard deviation of the mean

The main effect of stance type was significant in each of the five measures: APSD, *F*(1.57, 42.29) = 54.42, *p* < .001, ${\eta}_p^2$ = 0.67; AP range, *F*(1.61, 43.43) = 63.56, *p* < 0.001, ${\eta}_p^2$ = 0.70; MLSD, *F*(2.45, 66.22) = *p* < 0.001, ${\eta}_p^2$ = 0.93; ML range, *F*(2.41, 65.18) = 263.8, *p* < 0.001, ${\eta}_p^2$ = 0.91; overall mean velocity, *F*(3, 81) = 116.91, *p* < 0.001, ${\eta}_p^2$ = 0.81 (see Fig. 1). In almost all cases postural sway significantly increased between each stance type in the following order: bipedal < standard Romberg < tandem Romberg < unipedal. This trend repeated but did not reach significance in overall mean velocity for: bipedal vs. standard Romberg, tandem Romberg vs. unipedal, and in the MLSD data for tandem Romberg vs. unipedal. By exception, postural sway in ML range was greater in tandem Romberg vs. unipedal.

### Vision type

The main effect of vision type was significant in each of the five measures: APSD, *F*(1, 27) = 87.8, *p* < 0.001, ${\eta}_p^2$ = 0.77; AP range, *F*(1, 27) = 116.17, *p* < 0.001, ${\eta}_p^2$ = 0.81; MLSD, *F*(1, 27) = 782.6, *p* < 0.001, ${\eta}_p^2$ = 0.97, ML range; *F*(1, 27) = 430.76, *p* < 0.001, ${\eta}_p^2$ = 0.94; overall mean velocity, *F*(1, 27) = 160.73, *p* < 0.001, ${\eta}_p^2$ = 0.86. In each case postural s way was greater during EC compared to EO with the majority of the associated alpha levels < *p =* 0.001, those results that were above this threshold were non-significant (see Table 2).

**Table 2:** Mean (SD) measures, and mean difference (95% CI) for stance and vision type

Measure	Stance	Mean EO (SD)	Mean EC (SD)	Mean dif. (95% CI)
APSD (mm)	Bipedal	3.93 (1.34)	5.17 (2.37)	1.24 (0.69 to 1.8)
	Standard Romberg	4.66 (1.79)	5.97 (1.92)	1.31 (0.68 to 1.94)
	Tandem Romberg	6.83 (3.66)	10.01 (5.85)	3.18 (1.75 to 4.61)
	Unipedal	7.25 (2.33)	12.39 (2.5)	5.14 (4.41 to 5.89)
AP Range (mm)	Bipedal	22.19 (6.67)	28.94 (11.69)	6.75 (3.7 to 9.81)
	Standard Romberg	25.12 (8.24)	32.21 (8.86)	7.09 (4.53 to 9.66)
	Tandem Romberg	39.22 (18.63)	60.95 (36.09)	21.73 (12.43 to 31.02)
	Unipedal	42 (14.84)	76.38 (17.61)	34.38 (28.31 to 40.45)
MLSD (mm)	Bipedal	1.76 (0.8)	2.1 (1.01)	0.34 (0.12 to 0.56)
	Standard Romberg	4.45 (1.31)	5.67 (1.81)	1.22 (0.88 to 1.56)
	Tandem Romberg	5.41 (1.02)	10.31 (2.01)	4.9 (4.42 to 5.38)
	Unipedal	5.53 (1.12)	10.33 (1.39)	4.8 (4.46 to 5.15)
ML Range (mm)	Bipedal	11.99 (6.78)	13.55 (6.33)	1.56 (− 0.42 to 3.55)*
	Standard Romberg	23.33 (6.42)	31.08 (9.22)	7.75 (5.86 to 9.65)
	Tandem Romberg	29.83 (5.02)	51.63 (11.42)	21.8 (18.65 to 24.95)
	Unipedal	29.77 (5.12)	45.99 (6.37)	16.22 (14.41 to 18.02)
Mean Velocity (mm/s)	Bipedal	51.02 (14.85)	51.24 (14.58)	0.22 (−1.22 to 1.67)*
	Standard Romberg	52.89 (15.9)	53.75 (14.26)	0.86 (−1.1 to 2.84)*
	Tandem Romberg	71.71 (16.45)	92.92 (20.06)	21.21 (15.3 to 27.1)
	Unipedal	65.31 (12.83)	105.83 (21.41)	40.52 (34.22 to 46.83)

*AP* Anterior-posterior, *ML* Medial-lateral, *SD* Standard deviation, *EO* Eyes Open, *EC* Eyes Closed, *dif.* difference, *CI* confidence interval

*non-significant change

### Stance type * vision type

This two-way interaction was significant within each COP measure: APSD, *F*(2.01, 54.27) = 143, *p* < 0.001, ${\eta}_p^2$ = 0.46; AP range, *F*(1.57, 42.45) = 23.37, *p* < 0.001; ${\eta}_p^2$ = 0.46, MLSD, *F*(3, 81) = 211.91, *p* < 0.001, ${\eta}_p^2$ = 0.89; ML range, *F*(3, 81) = 66.48, *p* < 0.001, ${\eta}_p^2$ = 0.71); overall mean velocity, *F*(2.12, 57.27) = 85.23, *p* < 0.001, ${\eta}_p^2$ = 0.76. Post-hoc results reflected the general finding that while removing vision produced postural disturbances in all four stance types, this effect was stronger in those stances with inherently larger postural sway (i.e., unipedal > tandem Romberg > standard Romberg > bipedal). See Table 2. All other two-way and three-way interactions within each measure were not significant.

## Discussion

Compared to smooth insoles, textured insoles produced a statistically significant reduction in the standard deviation of anterior-posterior sway (3.08%; effect size = 0.14). While this main effect was significant when the data were collapsed across stance and vision type, the most pronounced impact was during bipedal standing with eyes closed; one of the least challenging and most natural stance types. The same trend was observed in the standard and tandem Romberg positions, but did not reach the level of significance, with no trends for unipedal standing. The textured insole had no significant effects on any of the other COP measures of interest. Presumably the intact postural control system in healthy young adults is adept at maintaining balance, making TI effects small in magnitude and difficult to observe in this group overall. The effect magnitudes we found in bipedal standing (10.5%), however, were stronger than those obtained previously for TIs in healthy young adults (6.82% [7]). Given the general importance of improving balance per se, in both healthy and clinical populations, we regard these isolated but significant findings as insightful for developing future interventions. In addition, a healthy young participant group was deemed necessary given the extended balance testing and their lack of pathologies, which allowed us to assess potential mechanisms of TIs effects on postural sway in multiple stance types.

The APSD variable is a key index of spatial variability [21, 22]. Reductions in this measure can specifically translate into improved maintenance of upright balance and a significant reduction in the prevalence of falls [23]. While TIs have previously been recommended for healthy young adults, based on improvements in ankle inversion/eversion in ballet dancers [24] and footballers [25], our results go further. We show positive TI effects in a variable associated with fall occurrence.

It is likely that introducing novel texture to the plantar surface of the feet enhanced afferent sensory input via the mechanoreceptors. Little is currently known, however, about the underlying mechanisms by which TIs effects occur. So far, two studies found TIs did not alter lower limb muscle activity (assessed using electromyography) during bipedal standing in healthy young [7] and older adults [3]. Other studies have shown, however, that human balance is controlled at least in part at a higher cortical level, rather than purely at a spinal level [17, 26]. Augmenting either an increase or alteration in afferent sensory information via TIs may therefore facilitate the estimation of error (with regards to the body’s position in space) undertaken at the cortical level [27], resulting in the balance improvements we observed in the present study. It will be important to investigate this proposal in future neuroimaging studies.

While we observed a clear linear increase in postural sway from bipedal to standard Romberg, to tandem Romberg, to unipedal across almost all variables, insole type did not significantly interact with stance type in any measure. In the APSD variable simple main effects revealed TIs reduced spatial variability in all but the unipedal stance. This suggests the alteration of afferent information via TIs during single-foot balance is insufficient for overcoming the inherent difficulties of this stance. Since participants completed 30s of balance more frequently in the tandem Romberg compared to unipedal stance (96.1% vs. 71.4%, respectively), we suggest tandem Romberg is more suitable for studying TI effects under challenging conditions.

Occluding vision significantly increased postural sway in each COP measure. Given the likelihood of ‘sensory reweighting’ during occluded vision (i.e., toward proprioceptive sources [16]) and since TIs most likely alter somatosensory input via the mechanoreceptors in the feet, it was surprising no interaction between vision and insole was found. Post-hoc analyses revealed, however, that TI effects were significant in the APSD variable during the bipedal stance with eyes closed, but only close-to-significant with eyes open (10.7% vs. 10.3% reductions for TI, respectively). These results support previous research showing greater TI effects during EC conditions [4–6]. They also align with the notion of sensory reweighting during occluded vision.

The significant two-way interaction in each COP measure revealed more pronounced effects for vision type when stance stability decreased. This points to a greater reliance on vision during more challenging stances. Accordingly, such sensory reweighting during EO should then diminish TI effects, which are presumably driven by somatosensory sources. While this result challenges the practical relevance of the eyes closed condition for some healthy and clinical populations, it forms a sound rationale for investigating TI effects in the partially sighted.

The present research adds to the literature advocating the use of TIs in healthy younger adults (c.f. [4, 7]), as evidenced by significantly improved postural control in APSD during bipedal balance. This index of spatial variability is associated with improvements in upright balance [21–23] and therefore could translate into a reduction in fall risk. It is, however, important to note we utilised only one texture type. It is therefore too early to rule out other textures, which may have differing degrees of effects on postural sway. In addition, the importance of assessing the mechanisms of such interventions (TIs) requires a healthy young group, as they are clear of any underlying pathologies and can undergo more rigorous testing procedures.

It is important to note that our findings are derived from a young healthy population, therefore the results cannot be fully extrapolated to inform on the balance abilities in those populations at greater risk of falls; such as older adults or clinical populations with known balance impairments. We recommend further work to be completed in clinical populations, such as people with either neurodegenerative diseases or sensory deficits. For example, individuals with loss of foot sensation show a greater reliance on and awareness of an altered somatosensory input [28]. Future studies should now investigate if balance can be similarly improved in such groups through TIs, particularly in those with visual impairment.

Research should also now investigate the longitudinal effects of TIs in healthy young adults and explore if these are associated with balance-related injury prevalence, for example, in sports and exercise settings. It will also be important to assess the time course of TI effects, which may dissipate through acclimatisation after an initial period of effectiveness. Future research into TI effectiveness during dynamic balance (i.e., gait) in sports and exercise will also be insightful, where the postural control demands are constantly changing. Finally, future studies should look to determine the underlying mechanisms by which these insoles effect postural sway. This could be accomplished by assessing exposure to TIs at a neuromuscular and cortical level. In summary, the positive results in the present research help clarify the benefits of TIs in healthy young adults across multiple stance types, paving the way for further investigations into the TI effectiveness for improving balance.

## Conclusions

Overall our results support TI use in healthy young adults for reducing postural sway measures. This is represented by a significant decrease in the APSD; an index of spatial variability, where a decrease is associated with improved balance. Placing a novel texture in the shoe presumably modulated somatosensory inputs arising from the soles of the feet. It is important to understand the underlying mechanisms by which TIs influence postural sway. To this end, utilising a healthy young adult group allows for the investigation of such mechanisms. Future research could investigate the potential underlying mechanisms of TI effects at a neuromuscular and cortical level, in healthy young adults.

## Abbreviations

AP: Anterior-posterior
APSD: Anterior-posterior standard deviation
COP: Centre of pressure
EC: Eyes closed
EO: Eyes open
ML: Medial-lateral
MLSD: Medial-lateral standard deviation
mm: Millimetre
mm/s: Millimetres per second
SI: Smooth insole
TI: Textured insole
